# Urban household food insecurity and pediatric malnutrition: evidence from the Divina Providencia Hospital in Luanda, Angola

**DOI:** 10.3389/fpubh.2025.1604713

**Published:** 2025-09-17

**Authors:** Andrea Scimone Carbone, Federico Roscioli, Matteo Mazziotta

**Affiliations:** ^1^Department of Economics, RomaTre University, Rome, Italy; ^2^Department of Economics and Finance, University of Rome Tor Vergata, Rome, Italy; ^3^Istat, Rome, Italy

**Keywords:** food security, poverty, malnutrition, capability approach, CART, Luanda, Angola

## Abstract

**Introduction:**

Food insecurity and poverty mechanisms often intertwine, negatively affecting the well-being of families and children, particularly those with acute malnutrition. In urban Angola, limited evidence exists on how socioeconomic deprivations influence household food security and treatment outcomes for malnourished children. Drawing on the Amartya Sen Capability Approach, this study investigates the determinants of household food consumption, weight gain during hospitalization, and outpatient treatment duration among children with acute malnutrition.

**Methods:**

We combined analyses from an original household survey of 84 families with hospital records from 1,259 children admitted to the Divina Providencia Hospital in Luanda between January 2019 and June 2021. Data included socioeconomic indicators, anthropometric measurements at admission and discharge, and treatment duration. Classification and Regression Trees (CART) were applied to identify key socioeconomic and clinical predictors across multiple dependent variables.

**Results:**

Lower household food consumption was observed when families could not afford at least 2.5 meals per day, had a monthly income below 30,000 Kwanzas, and when the mother’s education was less than 9.5 years. Anthropometric indicators at admission significantly influenced children’s ability to gain weight during hospitalization. Poverty and food insecurity were associated with longer outpatient treatment durations.

**Discussion:**

The findings highlight the effects of socioeconomic deprivations on food insecurity and recovery from acute malnutrition in children. Targeted interventions addressing low income, inadequate maternal education, and poor dietary access are essential to break the cycle of intergenerational poverty and malnutrition. Stronger policy implementation and integration of social protection with nutrition services are recommended to improve child health outcomes in peri-urban settings.

## 1 Introduction

Worldwide, 3 billion people cannot afford healthy diets, and about 811 million people suffered from hunger during the year 2020 (1). Furthermore, projections estimate that the prevalence of undernutrition in 2030 will match the value of the year 2005 (2). In Angola, severe and moderate food insecurity (FI) are estimated to hit nearly 50% of households of the country (3), and 1.59 million suffered from the food crisis in 2021 (4). FI implies inadequate dietary intake and is considered the leading risk of both malnutrition and poor health status of children (5). Food security (FS) in urban settings has received less attention from governments compared to rural areas in recent decades (6, 7). The presumptive positive effects of policies aiming at lowering food prices through increased availability (8) and the thought that urban diets tend to be more diverse compared to rural diets (9), possibly contributed to building the false myth of the ‘urban advantage’. Studies highlight that urban FS is primarily a function of food affordability through income derived from employment (10). In this paper, we consider the ability to be food secure to depend on several interlinked deprivations. Although in urban settings there is currently a knowledge gap on FS (11), some studies addressed the role of its social and economic components on diet appropriateness and child nutrition. In an urban hospital in the Democratic Republic of Congo poor breastfeeding practices, low average meals per day, family size, unemployment, and the level of mother education were found to be determinants of wasting in children (12). In Ecuador, a study highlighted that poor maternal mental health is detrimental to optimal child care and FS (13), and in urban areas of South Africa, healthy food was more expensive in places where retailers were in fewer numbers compared to rural localities (14). Worldwide, nearly half of the deaths of children under 5 years of age are due to malnutrition (15), and in Angola in 2020, 37.7% of children under 5 years of age were estimated to be stunted (82). Malnutrition still lacks a universally agreed definition (16), in this study, we rely on the definition provided by the ASPEN Board of Directors. Severe Acute Malnutrition (SAM) has three syndromes: edematous (Kwashiorkor), non-edematous (Marasma), and mixed form. All of them require medical intervention at the hospital level. Treatment of SAM can be divided into stabilization and rehabilitation phases (17). The stabilization phase aims to address clinical complications, such as dehydration, hypothermia, infections, diarrhea, and vomiting, among others (18). The rehabilitation phase follows clinical stabilization and aims to normalize body composition and growth rate. Typically, it involves scheduled nutritional reassessment meetings in the form of an outpatient treatment program (OTP) and the provision of food therapy (RUSF supplements). The consequences of malnutrition on well-being extend from the individual to the community level. At the individual level, it increases the direct risk of dying (19) through greater susceptibility to life-threatening opportunistic diseases (20). Long-term, food deprivation is detrimental to early child development goals (21) and to schooling achievements such as numeracy and literacy (22). At the community level, delayed physical and cognitive development implies lower access to employment in adulthood, poor economic conditions, and intergenerational transmission of poverty (23). Since a complete understanding of the mechanisms of death from malnutrition remains unclear (24), research connecting poverty, household FI, child malnutrition, hospitalization, and mortality is mandatory to inform policymakers about possible solutions to build an urban food system that ensures FS for all (25) capable of preventing the insurgence of SAM. In this regard, Tim Lang has long insisted on the need to frame food policies that address the interaction between the food system and its environmental and health outcomes (26). This study contributes to the literature on FS and malnutrition in periurban Luanda by combining information collected from different sources. Taking inspiration from Amartya Sen Capability Approach for food security assessments, the study investigates on how socioeconomic deprivations influence both the ability of families to be food secure and the ability of malnourished children to recover from acute malnutrition. In particular this study has a three-fold objective. Firstly, it aims to identify which socioeconomic factors at the municipal and household level affect the food consumption of a sample of families who regularly refer to Divina Providencia Hospital due to child malnutrition; Secondly, it analyzes how anthropometry at hospital admission influence the average gain weight during the stabilization phase at the hospital level; Thirdly, it addresses how poverty and FI affect the duration of treatment in the rehabilitation phase, after the children upgrade from the acute phase of malnutrition. The study combines original household data with hospital records compiled between January 2019 and December 2020. This study was designed to interview the caregivers of children affected by acute malnutrition with the aim of identifying which capabilities tend to determine food insecurity and consequently malnutrition in children. Such findings are of paramount importance to inform on targeting and priorization of the most vulnerable families, as well as to settle the basis of potential social protection policies. The remainder of the paper is structured as follows. Section 2 describes the framework of the research; Section 3 reports on the methods of data collection and the statistical approach used. The results and discussion are in Section 4, illustrating the deprivations that families share at the municipal level; the role that socioeconomic characteristics (SEC) play in determining the level of food consumption of families; the relationship between anthropometry, average gain weight (AGW), and mortality of children; and the influences of SEC on the time-length of the rehabilitation phase during outpatient treatment (OTP). Finally, in Section 5, our conclusions on poverty, FI and child malnutrition in Luanda are drawn. This paper challenges the conventional view that malnutrition is solely a function of caloric intake and energy requirements. It hypothesizes that deprivations constitutes the basis of multidimensional poverty and that consequently they play a determinant role in enhancing malnutrition. The authors also assume that productionism does not esnure food security for all. It hypothesizes that capabilities including being able to access to education, employment, health among others are critical determinants of household food security. Finally, this paper seeks to answer the following research question: how does the integration of the capability approach reshape the understanding of food insecurity and malnutrition at the household level?

## 2 Research framework

**Figure 1:**
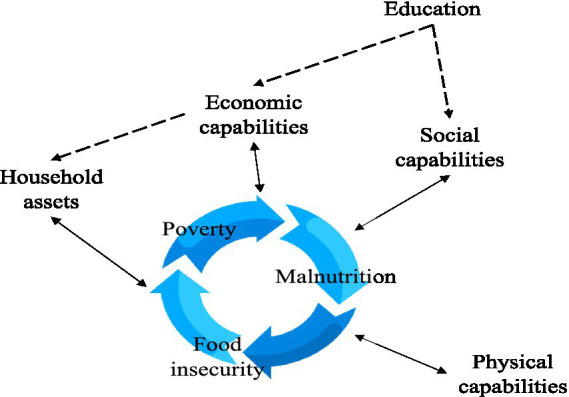
The logical framework of the research.

Figure 1 shows the logical framework used for data collection and analysis. It draws inspiration from the methodology of food security assessments based on the Amartya Sen capability approach proposed by Burchi and De Muro (27). Since the ability of a nation to provide food at the country level does not imply that all people will be able to secure it in a stable manner (28), the capability approach focuses on individuals and intends the concept of freedom as a function of the set of capabilities through which they may achieve their valued beings or doings. In particular, it considers income as a mean rather than an end of well-being, and it switches the focus from the command over food as intended by the income approach, to the wider range of capabilities that contribute to achieving the ability to be food secure, such as being educated or being in good health. The capability approach views food security as an area of study more inherent to well-being and human development research rather than under agriculture or nutrition. Several studies highlight the mechanisms through which deprivations affect FS at the household level. For example, uneducated parents may not be able to address the nutritional needs of newborns due to a lack of childcare knowledge (29). Additionally, since lower education is associated with higher fertility (30, 31), subsequent pregnancies could interrupt breastfeeding of the older child and induce early complementary feeding that affects the child’s nutritional status (32). Families unable to access employment could have a lower purchasing power to buy enough healthy food for everyone (33, 34). Households excluded from water and electricity systems might cook fewer meals per day due to volatility in the price of substitutive fuels (35) or due to the unavailability of water (36) while food scarcity could reduce the size of portions (37). Lastly, inefficient transportation systems could entangle food purchases from far-flung retailers (38). Consequently, poverty in economic and social capabilities might affect the ability to be food secure, well-nourished, and in good health.

**Figure 2:**
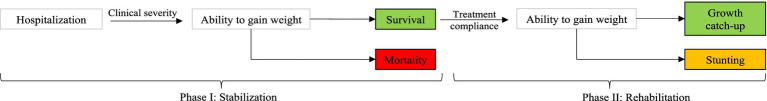
Severely malnourished children go through hospitalization where clinical conditions are stabilized. In case of survival, they are sent home where they carry out the rehabilitation phase.

As shown in Figure 2 malnutrition often requires hospitalization. The severity of the clinical picture in terms of low anthropometric values at admission to the hospital is crucial for determining the ability to reestablish homeostasis (39, 40). The outcome of hospitalization also depends on many individual genetic, hormonal, enzymatic, and bacteriological factors that were not possible to include in the study. However, the ability to gain weight (AGW) is generally considered a marker of resilience to malnutrition in clinical practice (41). Children who survive hospitalization are discharged from the hospital and continue their diet and medical therapy at home. Adherence to the dietary recommendations at this time is associated with success in treatment and growth gain (42).

## 3 Methodology

### 3.1 The data

The Divina Providencia Hospital in Luanda is located in the Kilamba Kiaxi Municipality, a peri-urban area of the city. Over the years, the hospital has emerged as a city-wide benchmark in the treatment of acute malnutrition, for both severe and moderate cases. Following the Angolan National Protocol for the Treatment of Acute Malnutrition, children with SAM are hospitalized to stabilize their clinical conditions. Subsequently, the children are transferred to the OTP where their nutritional status is periodically reassessed while receiving highly specialized ready-to-use therapeutic food. In this study, two different sources of data were analyzed. The registers data gather information of children hospitalized with SAM. A total of 1,699 children with SAMwere hospitalized at the Divina Providencia Hospital of Luanda between January 2019 and June 2021. After data cleaning, the final number of children included in the analysis is 1,259.^1^ The dataset is periodically compiled by the clerks of the hospitals and comprises 15 variables: the gender of the child, the anthropometric measurements on both the day of admission and discharge including weight, height and middle-upper arm circumeference (MUAC), the rate of weight gain and the gain in millimeters of the MUAC, the treatment duration, the outcome (dead / survived), the presence of edema and the area of residency of the family. Due to the hospital reporting requirements, the age of the children was aggregated in age groups: < 6 months, from 6 months to 59 months, and > 60 months. The main limitation of this data set is that there was no record of nutritional therapy administered to each child, nor if there were comorbidities. The average weight gain rate for each child was calculated with the following formula:


$$\frac{\left((\text{Weight}\;\mathrm{at}\;\text{discharged}\;(\mathrm{Kg})-\text{Weight}\;\mathrm{at}\;\text{Admission}\;(\mathrm{Kg})\right)}{(\text{Weight}\;\mathrm{at}\;\text{admission}\;{\left(\mathrm{Kg}\right)}^{*}\;\text{Days of Hospitalization}))\;*1000}$$

Since the registers did not include information about the socio-economic conditions of families, a survey was implemented to show how deprivations and lack of capabilities relate to the well-being and food consumption of families who referred to the hospital in August 2021 to seek hospital services. A sample of 84 families whose children were in the outpatient treatment program for malnutrition were interviewed in August 2021.

The hospital board granted formal authorization to conduct the survey. Data were collected through interviews conducted in the hospital’s nutrition ward during scheduled nutritional follow-up visits for children. Caregivers were recruited using a convenience sampling strategy based on their availability and willingness to participate and an original questionnaire was redacted and used to the scope of answering to the research question. Prior to each interview, the scope of the research was presented to the caregivers both in Portuguese and in local language. Also it was clearly specified that participation in the study was voluntary and that it would not affect the nutritional service, nor their right to receive food and the entitlements provided by the hospital. Verbal consent was asked to ensure caregivers with limited literacy or writing skills. A total of 84 families provided verbal consent to participate in the reasearch. The original questionnaire included quantitative and qualitative questions about SEC, anthropometric measurements of children, and weekly food consumption at the household level. By its nature, the capability approach requires a large informational basis to enable a comprehensive analysis of direct and sometimes hidden drivers of food security. Hence the reason of the authors to include in the questionnaire a range of multidimensional variables including education, literacy, age, family size, assets ownership, religious affiliations, income, health conditions, employment, nutritional, livelihoods and head of the household sex. This approach allows the study of food security to move beyond the “mechanicistic” perspectives of Income-based or the food availability approaches, to a broader area of human development studies. As a proxy indicator of food security we used an index of food consumption, computed by multiplying the frequency of weekly consumption of each food group by scores that underline the differences in the bromatological composition of food groups and then summed together to obtain a single value for each household (43). The indicator measures the food consumption of families in terms of quantity and quality. It has been used to assess food access (44) and has been validated to conduct in-depth food security assessments elsewhere (45). Our research is based on three main assumptions. First, food consumption is intended as a proxy measure for food security at the household level assuming intra-household distribution equity; second, we asked about food consumption at the household level assuming it to be positively associated with child food consumption (46). Third, we assumed that diet appropriateness is positively associated with the socioeconomic condition of households (47, 48).

### 3.2 The statistical methods

We used classification and regression trees (CART). Classification trees use recursive binary splitting based on the variables that have the least level of impurity (weighted Gini Index) in assigning samples to the right terminal node of the tree. Weighted Gini impurities are calculated for all independent variables: those having the least value of impurity represent the direct follower of the root.


$$\text{Gini impurity}\;(g)=1-{\left(\text{Probability of}\;\mathrm{yes}\right)}^{2}-{\left(\text{Probability of}\;\mathrm{no}\right)}^{2}$$

$$\text{Weighted Gini impurity}=g_{1}\cdot \frac{n\;\mathrm{lea}f_{1}}{N}+g_{2}\cdot \frac{n\;\mathrm{lea}f_{2}}{N}$$

Regression trees, instead, estimate the mean value of a quantitatively dependent outcome variable, given multiple independent factors. As well as classification trees, regression trees are built in a top-down direction. For choosing the root variable, each independent factor is used singularly to estimate the outcome variable through the process of least squares residuals. The variable that results in the minimum sum of squared residuals (RSS) is chosen as the top variable of the three. The same procedure is generated for the following splits. The tree stops when each leaf represents a minimum number of observations established *a priori*.

CART analysis usually produces models that are easy to analyze due to their tree-shaped output. Other studies have applied CART and regression analysis in malnutrition studies. Yin et al. have applied CART to develop a model for classifying the severity of malnutrition in cancer patients and accelerating pretreatment classification to achieve higher survival rates (49). CART are also likely to overfit the data due to extra ramifications. To reduce bias, fully grown trees have been pruned by controlling for the cost complexity parameter that minimizes the error rate of the prediction of each tree (50).

## 4 Results and discussion

### 4.1 Environmental deprivations: the characteristics of poverty at the municipal level of Luanda

**Figure 3:**
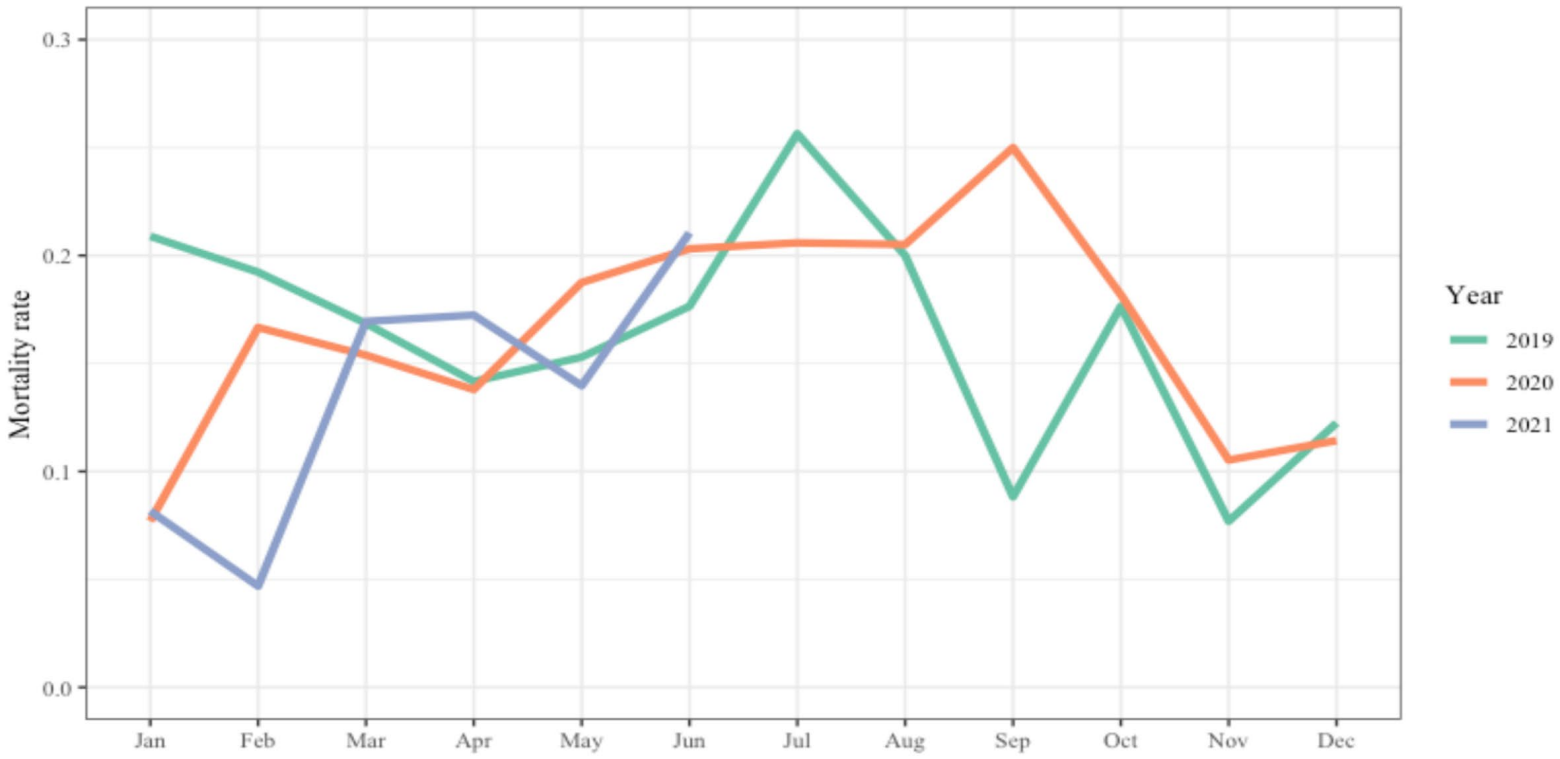
The graph shows the monthly rate of malnutrition lethality within the nutritional ward of the Hospital Divina Providencia of Luanda from January 2019 to June 2021.

Climate conditions in Luanda must be considered when studying malnutrition. In fact, The weather in Luanda is dry from May to September and rainy from October to April (51) and Figure 3 shows that the level of malnutrition mortality in the Divina Providencia hospital peaked during the dry season in both 2019 and 2020, thus providing a connection between household food security (possibly due to the lack of water for agricultural use) and higher levels of malnutrition mortality. In 2020, the hospital did not accept new admissions for several weeks from February to March due to Covid-19 restrictions. However, children with SAM who were admitted to the hospital prior to February were allowed to continue treatment within the hospital. This partially explains why in 2020 the mortality peak was reached in the month of September, when families were finally allowed to seek nutritional help at the hospital.

Since the survey was implemented in August, during the dry season, analyzing how deprivations affect households in different areas is of paramount importance. To the scope, we applied CART analysis to our survey data. The hospital admits children from five main Municipalities namely Belas, Luanda, Kilamba Kiaxi, Talatona and Viana. These geographic areas were set as the dependent variable and all SECs as independent variables. SEC includes information about both the mother of the child and the head of the household, including age, education, literacy, and employment; it also includes information about the size of the family, the rooms available, the type of material for construction, income, access to water and electricity, the cooking fuel, the number of meals per day, the food expenditure, the place from where they usually buy the food, the month of interruption of breastfeeding of the malnourished child, the index of food consumption, and a series of assets owned by the family such as the fridge, the cell phone, the bicycle, the television, and the radio.

**Figure 4:**
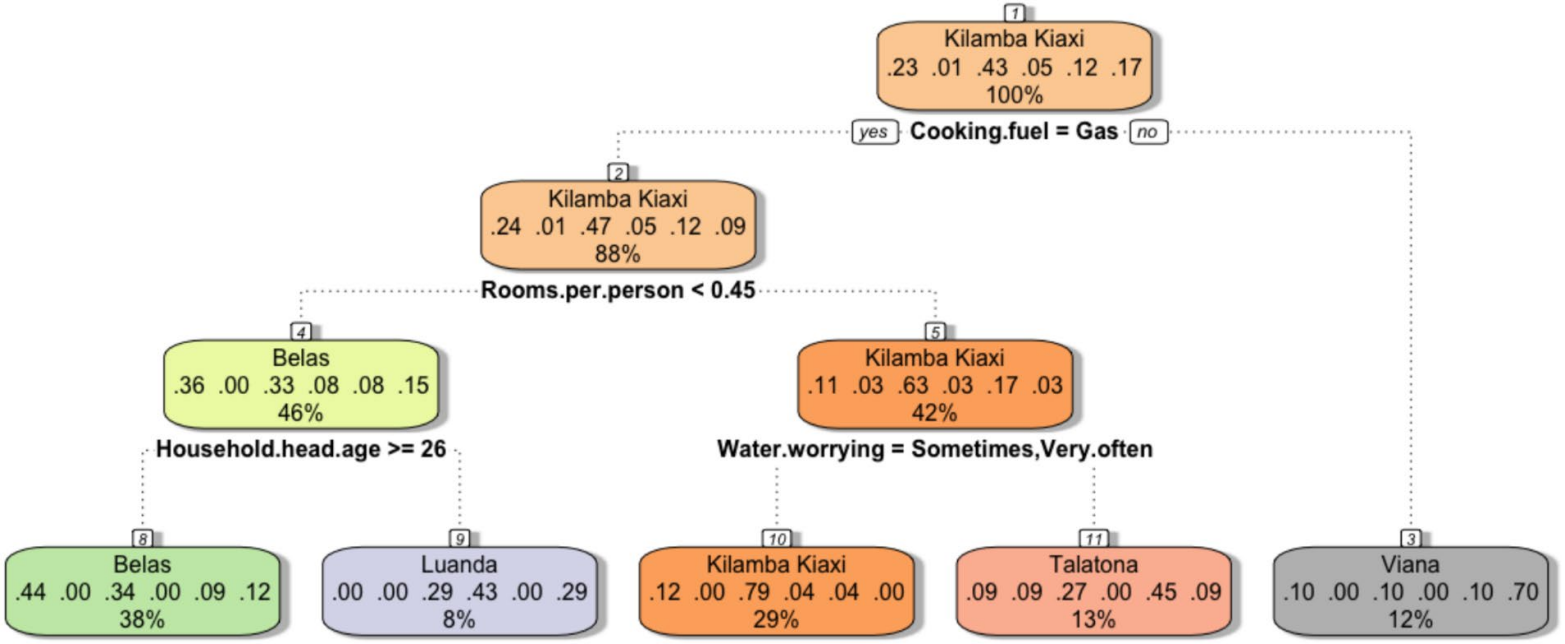
Classification tree built from survey data. Municipalities constitute the dependent factor, while the SEC of the families are the independent variables. Terminal leaves represent the percentage of families who arrive at the hospital from each municipality given the characteristics of each node.

By sampling, all the families interviewed shared the problem of child malnutrition. Figure 4 classifies families according to household characteristics and deprivations that they more frequently experience in the municipality where they live. Each node also provides the estimated percentage of the families who belong to each municipality in each split. The root node (the box at the top of the tree) provides the approximated percentages of families sampled per municipality: Belas (23%), Cacuaco (1%), Kilamba Kiaxi (43%), Luanda (5%), Talatona (12%) and Viana (17%). Thus, the majority of families tend to refer to the hospital from Kilamba Kiaxi, the municipality where the hospital is located. At each split the tree provides different percentages referring to each Municipality. For example, the first split identified the lack of a gas system as the best classifier of the entire survey sample. In this case, families living in Viana seem to be affected the most with a probability of 70% (gray box). The second split only applies to families that have access to the gas system. Families from Belas have a 44% probability of having a room/person ratio smaller than 0.45, while families from Kilamba Kiaxi have apparently better housing conditions. Furthermore, in Luanda, families seem to live in similar housing conditions as families in Belas, while they tend to have heads of household of different age groups. On the other hand, Kilamba Kiaxi families have lower gas systems and housing problems, but families living there have a 79% probability of experiencing anxiety in relation to the availability of water. The lack of a gas system could affect the ability to cook safely for entire communities. In fact, relying on substitutive fuels for cooking, such as wood or charcoal, can cause major health problems such as burnt and respiratory diseases (52). Furthermore, the price volatility of such materials can undermine the ability of families to prepare enough food (53). Families having limited access to fuel may exclude long-cooking ingredients from their diet and therefore limit their weekly food intake (54, 55). In Luanda, close to three quarters of urban citizens live in informal settlements called ‘musseques’ where house overcrowding is a spreading phenomenon (56), and can double the probability of cutting down the size of children’s meals (57). Furthermore, mental health markers such as anxiety and depression are known to increase when families experience water insecurity (58). Studies highlight that despite recent government actions, only 50.3% of the peri-urban population attain the minimum 40 liters per day of water consumption (59), which implies serious difficulties in food preparation. On the one hand, the informal system of water provision was seen as able to provide water to areas that are not secured by the formal system. This implies that quality controls are rarely or never conducted. Water quality has been associated with the suffering of environmental enteric dysfunction (EED), leading to intestinal inflammation, malabsorption, and therefore malnutrition, especially in children of overcrowded households (60). Furthermore, Mkupete et al. showed that unequal access to water and provision implied unequal child growth levels in Tanzania (61).

**Figure 5:**
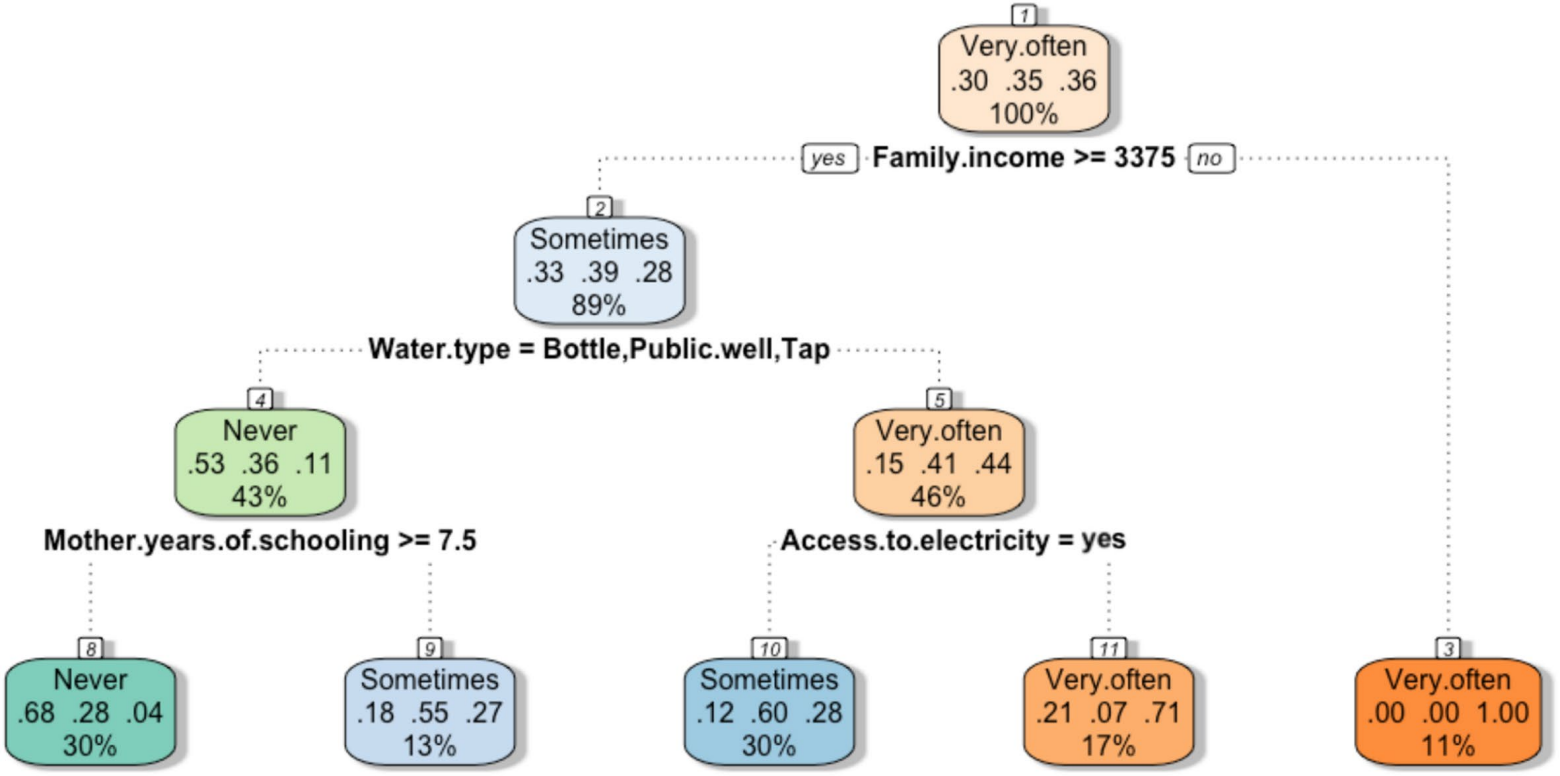
Classification tree. The feeling of anxiety in relation to water availability was established as a dependent variable, while the SEC characteristics of the families constitute the independent variables. The terminal leaves represent the probabilities that families feel worried about water security given the characteristics of each node.

The CART in Figure 5 provides an analysis of the issue of water security in Luanda. It shows that income, buying tanks/cisterns of water (which in Luanda implies depending on the informal water system), mother education, and inaccessibility to the electrical systems are the capabilities that affect to the probability of households experiencing worry about water unavailability. Families that earn less than 3,375 Kwanzas (7.5 $) per week (11% of the sample) are shown to experience anxiety in relation to water, due to lack of purchasing power even against the informal market. Additionally, if families that earn more than 3,375 Kwanzas per week are required to rely on the informal water supply system and, at the same time, have no access to electricity (17% of the sample) they will experience anxiety in relation to water. On the other hand, families who can rely on tap water or public wells or buy bottles of water experience significantly lower stress in relation to water availability. Another study highlights the mechanisms of interdependency between water and energy security in determining FS (62).

### 4.2 The effects of multidimensional poverty on households food consumption

**Figure 6:**
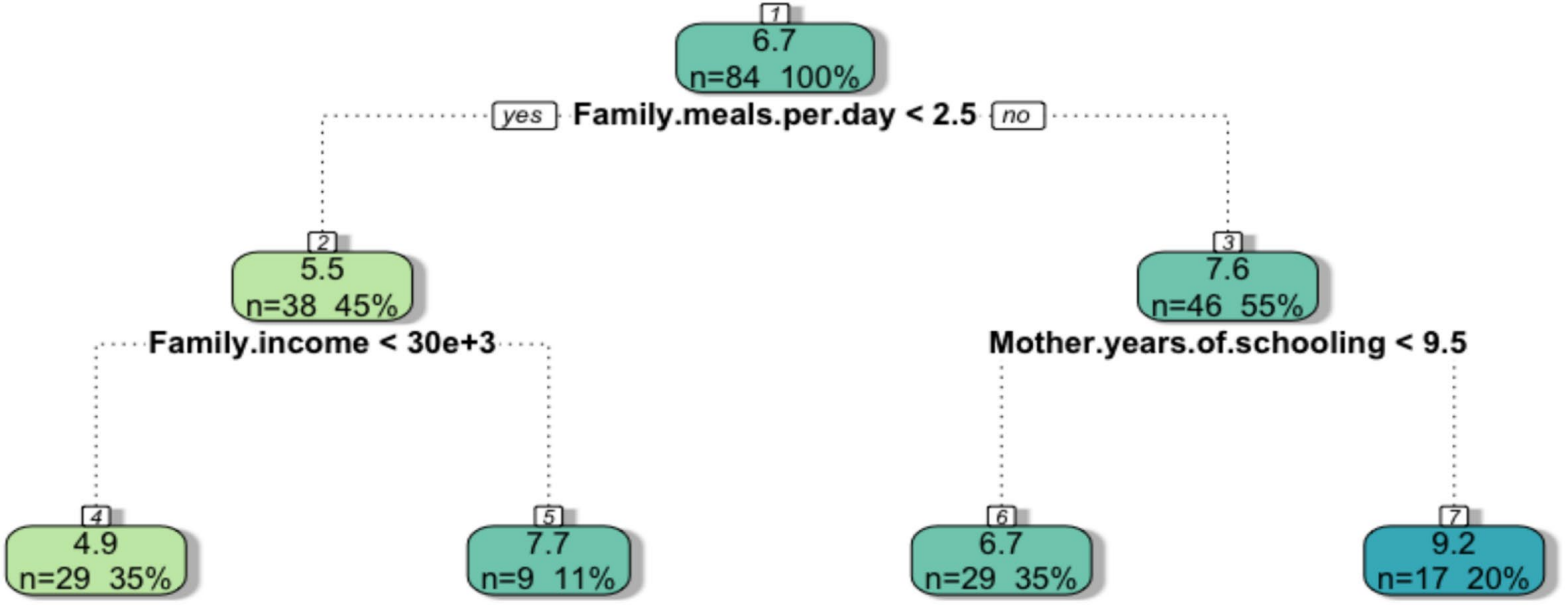
The regression tree results from applying the CART analysis to the survey data. The index of food consumption was set as the dependent variable, while the SEC of the families was prompted as independent variables. The terminal leaves represent the average index of food consumption of the families given determined characteristics at the household level [Regression trees read following the same logic as classification trees. Each box gives the average estimates of the outcome (in this case food consumption) depending on the conditions that at each level minimize the sum of the square residuals (RSS)].

To study the relationship between household characteristics and level of food consumption, we built a regression tree (Figure 6) setting the index of food consumption as the dependent variable and the SEC variables as independent variables. The index of food consumption ranges from 0 to 15 indicating increasing diet appropriateness.

Each box shows the change in the average index of food consumption of families depending on SEC and gives information on the number and percentage of families that are included in each split. Depending on the number of meals to which families have access (lower or greater than 2.5 per day), the score decreases to 5.5 or increases to 7.6. Therefore, Figure 6 highlights that the daily frequency of meals is a proxy for the weekly food consumption score. In addition, family income and maternal education resulted as factors that further differentiate the levels of household food consumption. In particular, families earning less than 30.000 Kwanzas are classified as having the lowest level of FS (4.9), while a level of mother education greater than 9.5 years implies the highest FS score (9.2). These results are in line with other studies in the literature. Several studies consider; several studies consider 3 meals per day as a conventional threshold of FS both in Zambia and Kenya (53, 63). Furthermore, other scholars found that the ability of families to purchase food was related to the lack of jobs and income in urban areas in Yemen (64). The positive relationship between maternal education and the levels of food consumption detected here has also been highlighted in other studies in which educated women from Burkina Faso, Mali, Niger and Chad had fewer children (65) and ultimately experienced fewer malnutrition events (66). Berlie et al. found that the educational level of the household is a strong determinant of FS in Ethiopia (6) while specifically through CART, the level of schooling of parents was found to be a strong indicator in classifying the nutritional status of children in Bangladesh and Cambodia (67, 68). We must recognize that all the households interviewed experienced child malnutrition, thus we cannot infer the causes of it.

### 4.3 Malnutrition, hospitalization and physical capabilities: the influence of anthropometry on weight gain and mortality

In this section, we connect the two data sources used in this study. Since the hospital register data did not contain information on the socioeconomic characteristics of families, we assume that temporary anthropometric wasting of the hospitalized children reflects the multidimensional poverty mechanisms as discussed in the earlier section. Indeed the findings from the survey data framed the inability to access enough meals per day, the inability to access to enough income and mother education as main deprivations that affected food security in the sample of families interviewed. Other studies have highlighted how malnutrition indicators can gage food system policies’ capacities to ensure the ability of a society to be food secure, avoiding the need for intricated monetary conversions (69). Also, the mere existence of hospital facilities in Luanda collecting data on the survival of malnourished children should already sound alarming in terms of urban food system dysfunctionality. However, we find it useful to show how data can shed light on nutritional patterns. Indeed, data-driven methods might reveal patterns that can complement policy formulation (70) including those aimed at eradicating malnutrition. In this case, we use a regression tree to explore how the severity of malnutrition indicators at hospital admission influence children’s ability to gain weight during nutritional treatment and consequently the survival rate. We set the AGW as the dependent variable while the anthropometry of children at admission to the hospital including weight, height, MUAC, together with the treatment outcome (survived/dead), gender, age, and the Municipality of provenance were set as independent variables.

**Figure 7:**
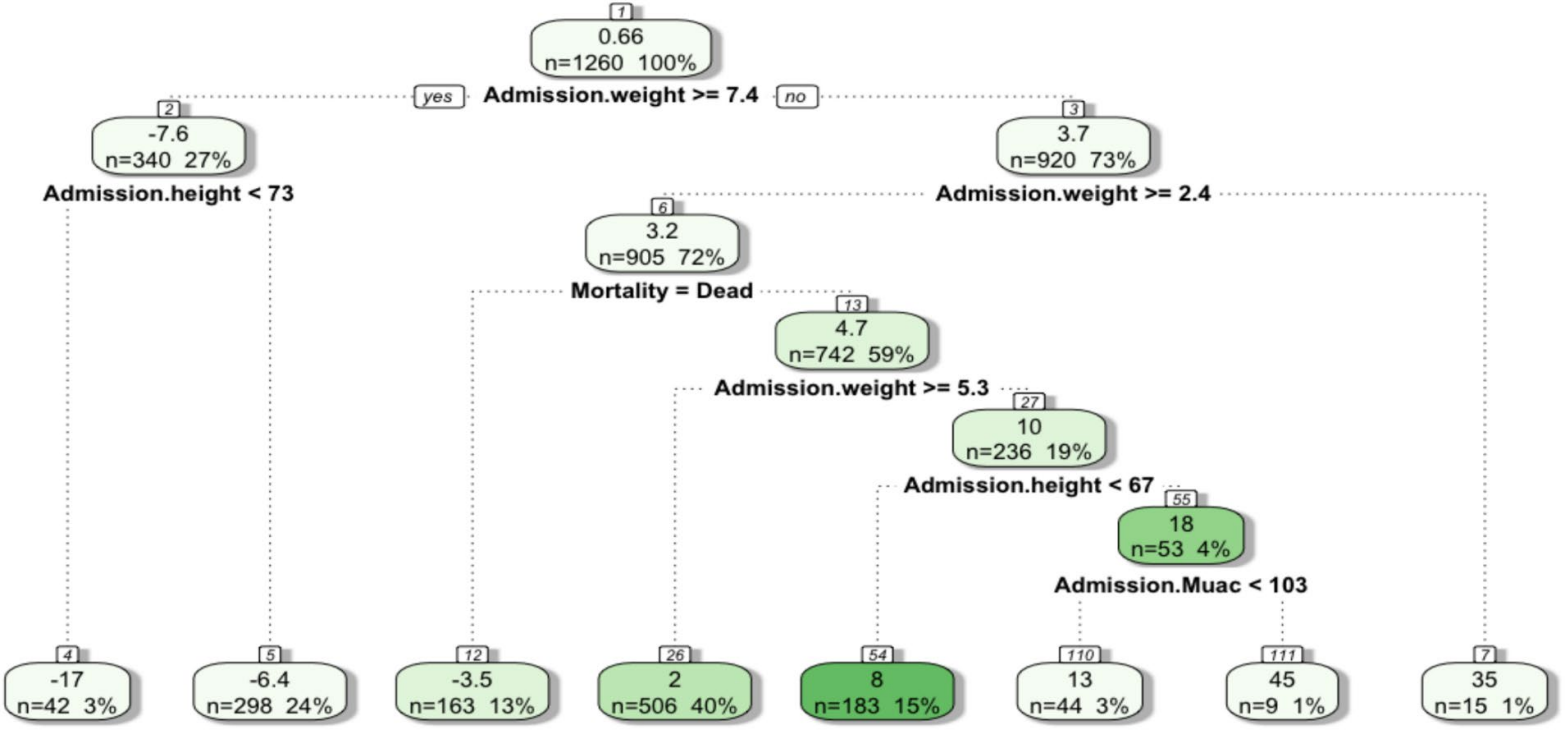
The average daily AGW was set as the dependent variable, while the anthropometric values at admission to the hospital, sex, age, municipality and outcome mortality/survival were set as independent variables. The terminal leaves of the tree represent the average daily AGW of the children given the features provided by each node.

The final leaves of the regression tree in Figure 7 illustrate different values of AGW during hospitalization, depending on the anthropometry of the children at admission. In particular, it estimates that the children who died (13% of the average estimated) weighed between 2.4 and 7.4 kg and accounted for a loss of weight averaging −3.5 g / kg / day during hospitalization. On the other hand, survivors tended to gain at least 2 g / kg / day.^2^ From the tree, the AGW of children varies greatly depending on the MUAC at admission, highlighting that a lower MUAC implies a lower gain weight during an average length of stay (LOS) of ~8 days (not shown). In another study conducted in Cunene (Angola), the AGW of malnourished children varied from 0 to 20 g/kg/day with a LOS of 7.9 days and a recorded death rate of 11.4% (71) highlighting a continuum among Angolan hospitals. In Egypt, the mean AGW was detected to be 10.4 g/kg/day with a LOS of 15.47 days (41), while in Ghana a weight gain per diem of 28 g / kg was measured during a LOS of 8 days (72). Differences in AGW could be due to varying clinical conditions at admission to the hospital. HIV, EED, diarrhea, or pneumonia might be the primary cause of weight loss (73).

**Figure 8:**
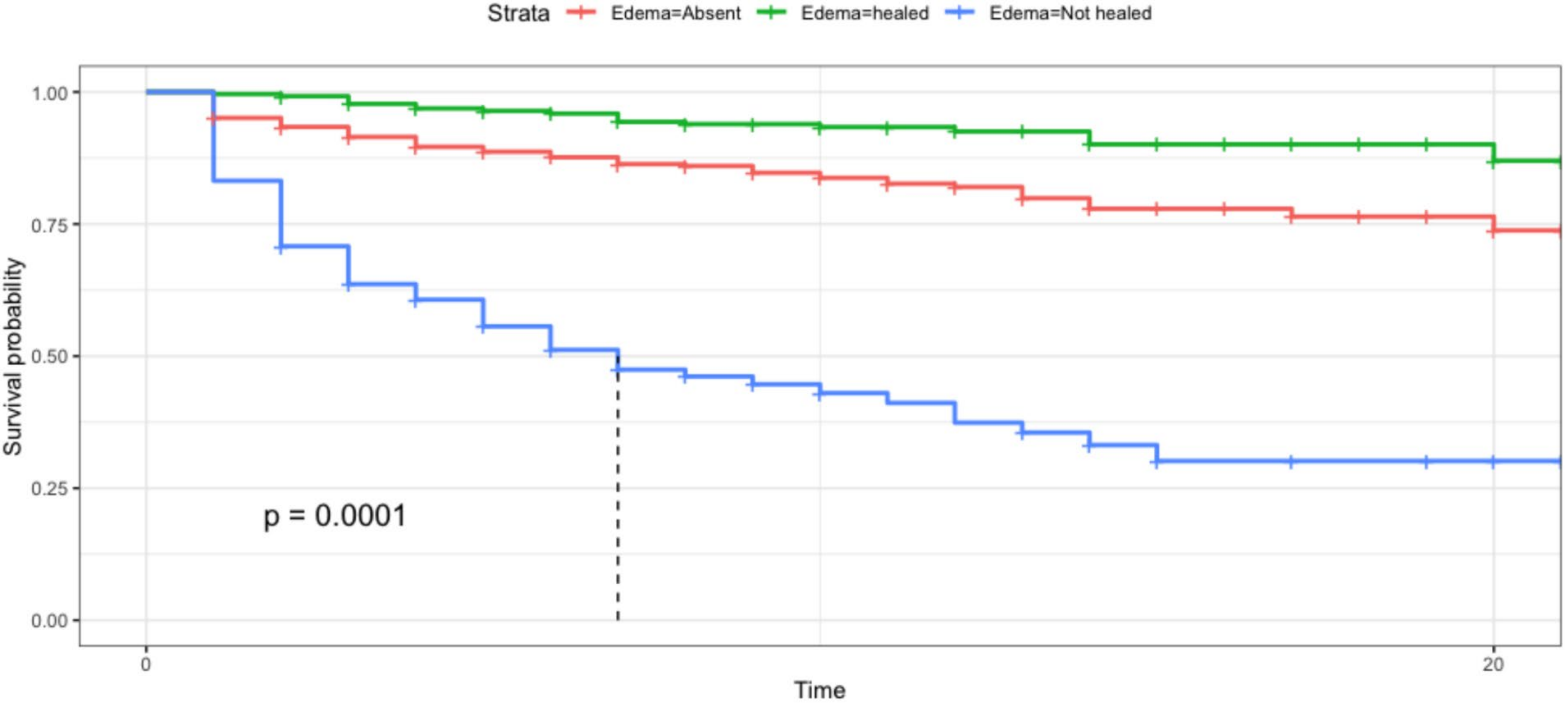
The Kaplan–Meier survival curve shows significant differences in the survival rates of children depending on the presence of edema along with the hospitalization for malnutrition. The x-axis shows the time of hospitalization in days, the y-xis shows the survival probability decreasing depending on time and the type of malnutrition.s.

In our data, no other symptoms other than edema were recorded in the registers. Nevertheless, the survival curve in Figure 8 shows that edema is a significant risk factor for mortality (74). Trends show different survival rates depending on the persistence of edema during each day of hospitalization. Unhealed edema on day 8 of hospitalization reduces survival probabilities to <50%. The exact biological pathway between mortality and complicated SAM has not been established and was not the objective of this study. However, it is relevant to note that prolonged starvation is associated with a wide range of metabolic changes (24) that are difficult to measure in daily practice in hospitals in developing countries.

### 4.4 The influence of deprivations on the duration of the outpatient treatment

**Figure 9:**
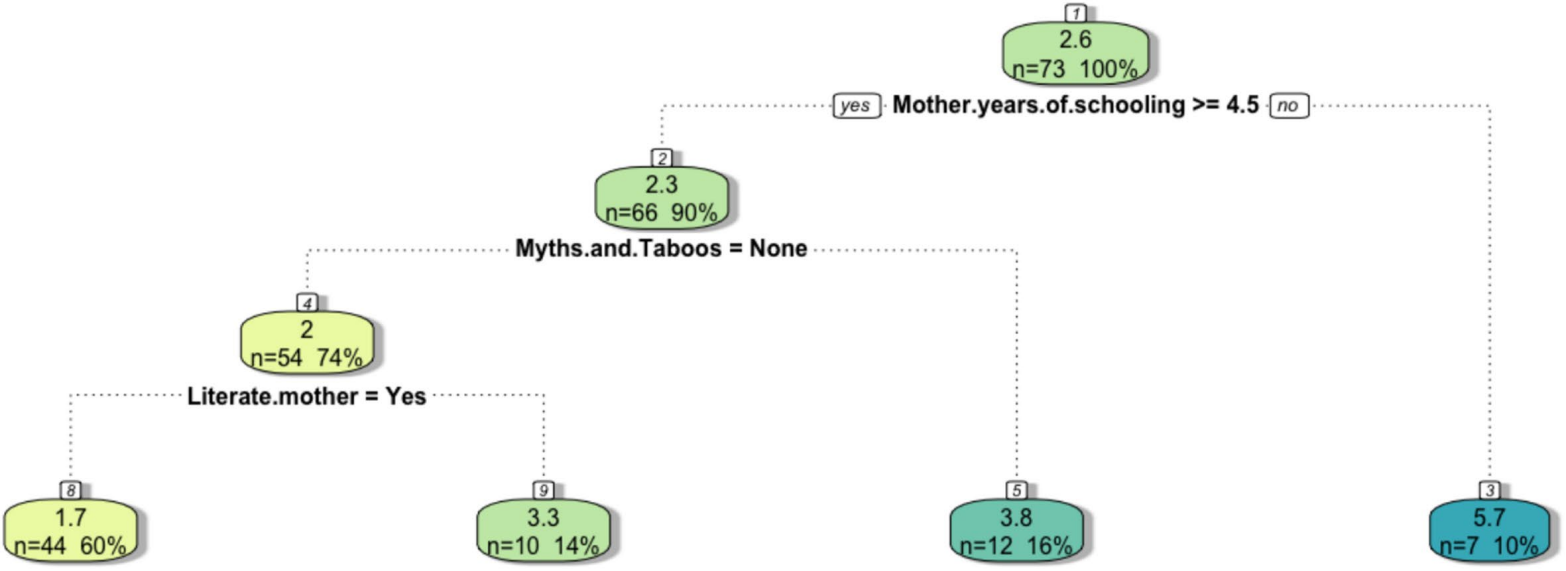
The regression tree infers the length of treatment of the rehabilitation phase. The final leaves represent the average duration of this period of malnutrition treatment given the conditions highlighted in the tree splits.

After hospitalization, the children begin the outpatient therapeutic care program (OTP). To identify household-level deprivations that may extend the length of OTP treatment and hinder timely recovery, the regression tree in Figure 9 was built with the duration of the treatment as the dependent variable while all SEC were set as independent variables. The tree shows that the levels of schooling, myths and taboos about food, and the literacy of the mother are determine separate degrees of duration of the treatment. The partition showed in Figure 7 highlights that maternal education is the most influential independent variable associated with the duration of the OTP treatment. Children whose mothers have more than 4.5 years of schooling tend to have a shorter treatment duration, averaging 2.3 months, compared to 5.7 months for children whose mothers have less than 4.5 years of schooling. In the group of children whose mothers had better access to school, the absence of cultural / religious beliefs is another influencial determinant of the duration of OTP of their children. In this case, the average treatemt varies from 2 months when families do not have any belief that might influence food consumption to 3.8 months on average when the families do belong to religious group who predicate abstinence from certain foods. Furtherly, when the mother lacks the ability to read and write the average duration of treatment increase from 1.1 months on average to 3.3 months on average. This analyses shows that deprivations such as low mother education and literacy as well as myths and taboos have a layered influence in determining the ability of the child to recover from malnutrition during OTP. This analysis also speculate that the nexus between mother education and the duration of treatment might be similar to the link highlighted in Figure 6 that connect slow mother education to low food consumption at the household level. Indeed, mother illiteracy has been associated with children underweight (75), stunting (76), worse Infant and Young Child Feeding Practices (77) and as one of the most important factors in combating malnutrition in at least three countries such as Ghana, Nigeria and Ivory Coast (78). Likewise, we argue that food restrictions imposed by religious or traditional beliefs may undermine children’s ability to recover, thereby extending the duration of treatment (79).

## 5 Conclusion

This article analyzed data collected in the malnutrition ward of the Divina Providencia Hospital of Luanda for a three-fold purpose. Inspired by Amartya Sen’s capability approach, this study aimed to assess the relationship between social and economic deprivations and family food consumption; to analyze how anthropometry at hospital admission influence childrens’ ability to gain weightt; to verify the link between household socioeconomic deprivations and the duration of the rehabilitation outpatient treatment. This article combines primary and secondary sources of data. In particular, we submitted a survey to a sample of 84 families in the hospital ward that directly deal with severe acute malnutrition and complemented that information with the hospitalization registers kept by the medical staff accounting for 1,259 children. Through classification and regression tree analysis, we firstly detected that food insecurity affect the well-being of families differently in different Municipalites. Deprivations such as the lack of access to cooking gas, household overcrowding, and the perception of water insecurity resulted as key socioecomic capabilities lacking in most of the Municipalities. These overarching deprivations resulted to be intertwined with idiosyncratic poverty at the household level. The ability to cook sufficient meals per day, the monthly income, and mothers’ education were identified as the main estimators of fluctuating levels of food consumption. Data from the hospital registers show that both anthropometric indicators at admission and the presence of edema are associated with the ability of children to gain weight, during the stabilization phase. Finally, we show that mother’ illiteracy and lack of education influence the duration of the outpatient therapeutic care. In conclusion, food insecurity in urban areas is a multidimensional phenomenon shaped by interlinked determinants such as lack of access to school and education, access to food and access to income. At clinical level, this study reinforces that malnutrition is a life-threatening condition that should be prevented rather than treated. It emphasizes the critical role that dietitians hold in assessing, preventing and treating malnutrition and their responsibility in strengthening the knowledge and the ability of caregivers in child feeding practices.

## 6 Policy implications

In such a complex environment, policies must go beyond one-dimensional systematic solutions and embrace a systemic human rights-based approach to food security. This requires acknowledging that food insecurity is not merely a matter of insufficient food production or importation, but rather the outcome of intersecting social, economic, and political deprivations. To this end, policy frameworks should, adopt a paradigm rooted in bottom-up food sovereignty aiming to the democratization of the food system, supporting local empowerment, sustainable agricultural practices and respect of food preferences. Despite the formulation of the National Food and Nutrition Security strategy (ENSAN) redacted in March 2009 (80), this study highlights that food insecurity and malnutrition still affect the well-being of families in Luanda. Integrating the capability approach into policy design and implementation can help ensure that interventions target the real constraints on people’s freedoms to live healthy, well-nourished lives while also enhance local food systems and protection of communities.

## 7 Limitations

This study is not exempt from limitations. First, we are not in a causal inference scheme due to the lack of available data and the ethical impossibility of imposing such a scheme. Second, our analysis is based on a limited sample of people due to the peculiar section of the hospital in which we collected the data. Third, the lack of information on possible comorbidities that affect children during hospitalization with SAM does not provide information about the cycle of infection-malnutrition.

## Data Availability

The datasets presented in this article are not readily available because it is hospital data containing the personal information of children and families. Requests to access the datasets should be directed to the corresponding author.

## Footnotes

^1^Duplicated data of children that were hospitalized more than once during the 2 years under study were removed, their reduced number did not allow for conducting a time-series analysis.

^2^WHO defines the AGW during hospitalization as poor (< 5 g/Kg/day) moderate (between 5 and 10 g/Kg/day) and optimal (> 10 g/Kg/day) (81).
